# Hepatic connective tissue growth factor expression and regulation differ between non-steatotic and non-alcoholic steatotic livers from brain-dead donor

**DOI:** 10.1038/s41598-021-83516-2

**Published:** 2021-02-16

**Authors:** Dong-Jing Yang, Ji-Hua Shi, Zong-Ping Xia, Wen-Zhi Guo, Mohammed Shakil Ahmed, Shui-Jun Zhang

**Affiliations:** 1 Department of Hepatobiliary and Pancreatic Surgery, Henan Key Laboratory of Digestive Organ Transplantation and Zhengzhou Key Laboratory for HPB Diseases and Organ Transplantation, The First Affiliated Hospital of Zhengzhou University, Zhengzhou University, Zhengzhou, China; 2 Translational Medicine Center, The First Affiliated Hospital of Zhengzhou University, Zhengzhou University, Zhengzhou, China; 3 Institute for Surgical Research, Department of Cardiology and Center for Heart Failure Research, Oslo University Hospital-Rikshospitalet and University of Oslo, Oslo, Norway

**Keywords:** Liver, Molecular medicine

## Abstract

Accurate evaluation of liver steatosis is required from brain-dead donors (BDDs) with nonalcoholic fatty liver disease (NAFLD). Our purposes were to investigate expression and regulation of connective tissue growth factor (CTGF) expression in livers from human and rat after brain death, and further evaluate its potential application. NAFLD and brain death models were established in rats. LX2 cells were cultured under hypoxia/reoxygenation. CTGF protein and mRNA levels were measured in liver samples from BDDs of human and rat by immunohistochemistry and reverse transcription-quantitative polymerase chain reaction. YAP-regulated CTGF expression was investigated in LX2 cells via YAP small interfering RNA and Verteporfin treatment. Blood CTGF level from BDDs was measured by enzyme-linked immunosorbent assay. After brain death, CTGF, transforming growth factor-β and YAP were overexpressed in non-alcoholic steatotic liver, whereas CTGF was downregulated in non-steatotic liver. Time-series analysis revealed that CTGF and YAP expression was comparable, as confirmed by inhibited YAP expression in LX2 cells. CTGF level and NAFLD activity were linearly correlated. CTGF expression and regulation differ between non-steatosis and nonalcoholic steatosis livers from BDDs. CTGF may be an important factor to evaluate graft quality from BDDs with NAFLD.

## Introduction

Donor shortage and consequent increases in waiting list mortality for liver transplantation have pushed clinicians to utilize extended-criteria liver graft from the brain-dead donors, which can lead to a suboptimal allograft outcome^1,2^. Nonalcoholic fatty liver disease (NAFLD) is a frequent contributor to suboptimal allografts from extended-criteria donors^3^. NAFLD represents a spectrum of diseases characterized by the excess accumulation of triglyceride within the liver, ranging in severity from reversible simple hepatic steatosis to progressive non-alcoholic steatohepatitis (NASH) and liver cirrhosis. As the prevalence of hepatic steatosis is increasing globally, it is expected that a higher proportion of potential donors worldwide will have liver steatosis, thus influencing the quality of grafts^4^. Hepatic steatosis, inflammation, and fibrosis constitute the major risk factors of impaired liver graft function and predict liver-related mortality in patients with NAFLD^5–7^, which requires careful consideration during graft evaluation. Few study has investigated the impact factors and the underlying mechanism of liver steatosis under acute stress of brain death before organ procurement^8^.

Since NAFLD is usually a silent disease with few or no typical symptoms, accurate evaluation of liver steatosis in potential donors is required. During liver graft procurement, assessment of steatosis through visual inspection has a positive predictive value of less than 20% for mild steatosis and only reaches up to 70% for severe steatosis^9^. Ultrasonography, computed tomography, and magnetic resonance imaging are useful modalities to detect NAFLD non-invasively; however, these assessments only provide qualitative information. Liver biopsy and histopathological changes of the potential liver graft remain the gold standard for a diagnosis of NAFLD and grading liver disease severity^10^. However, liver biopsy is an invasive procedure with potential adverse effects. Moreover, the prevalence of biopsy-proven NAFLD among potential donors was reported to range from 15 to 53%, which disqualified 3–21% of potential liver grafts^11^. The interpretation of NAFLD, and consequently graft usability, may be erroneous due to the heterogeneous distribution of hepatic steatosis and liver fibrosis. Steatosis factors may cause cellular injury, inflammation, and fibrosis through oxidative stress, mitochondrial dysfunction, endoplasmic reticulum stress, iron accumulation, apoptosis, and production of adipocytokines, which have been selected as clinical diagnosis and scoring biomarkers to assess the steatosis degree and NAFLD activity^12,13^; however, these markers have not been validated for graft evaluation.

Connective tissue growth factor (CTGF) expression in hepatic stellate cells (HSCs) and liver endothelial cells correlates with the extent of acute liver injury, and upregulated CTGF expression was proposed as a central pathway contributing to hepatic stellate cells (HSCs) activation during liver chromic inflammation and fibrosis^14,15^. Most livers for transplantation are procured from brain-dead donors. The irreversible brain injury causes hemodynamic instability and hormonal disturbance, which could trigger acute liver injury^16^ and thus complicate the pathological process of NAFLD activity^17^. Specifically, acute inflammation, steatosis, and fibrosis caused by NAFLD might aggravate the activation of HSCs and lead to CTGF upregulation in the non-alcoholic fatty liver from the brain-dead donor^14,15,17^. The expression and regulation of hepatic CTGF in brain-dead donors have not yet been studied.

To investigate the overall effect of brain death on intrahepatic gene expression, we applied RNA-sequencing to investigate hepatic transcript level of non-steatotic liver from brain-dead donors. However, the results indicated that CTGF expression was down-regulated in non-steatotic liver from brain-dead donors^18^. Thereby, we hypothesized that hepatic CTGF expression and its regulation after brain death might be different between non-steatotic liver and non-alcoholic steatotic liver. To test this hypothesis, we investigated the expression pattern and regulation mechanism of CTGF in non-steatotic liver and non-alcoholic steatotic liver from brain-dead donors in human and rats, and explore the potential clinical application of CTGF.

## Materials and methods

### Animal models and study protocol

The animal experiments in this study conformed to the ARRIVE (Animal Research: Reporting of In Vivo Experiments) guideline and were approved by the Institutional Animal Ethics Committee of Zhengzhou University (No. 2019-KY-019). All methods were performed in accordance with the relevant guidelines and regulations. The male Lewis rats were purchased from the Beijing Vital River Laboratory Animal Technology Co., Ltd. (Beijing, China). All the 24 rats were housed in laminar flow cabinets under specific pathogen-free conditions at room temperature with a 12 h light/dark cycle, with food and water available ad libitum.

The model of NAFLD was established by feeding rats with the high fat diet^19^. The non-alcoholic steatotic liver group (n = 10) was fed with a high-fat and high-cholesterol diet that consisted of 85% standard diet, 13% lard oil and 2% cholesterol for 4 weeks. The non-steatotic liver group (n = 30) was fed with a standard diet before establishment of brain death.

In the rats from non-alcoholic steatotic and non-steatotic liver groups, brain death was established by increasing intracranial pressure and was further kept over a follow-up period^20^. To evaluate the dynamic change after brain death, a number of 5 rats in the standard diet group were euthanized and liver samples were collected at 0 h, 1 h, 2 h, 4 h and 6 h after brain death. Five rats in non-alcoholic steatotic liver group were euthanized and liver samples were collected before brain death and at 6 h after brain death, respectively (Supplementary Fig. 1).

**Figure 1:**
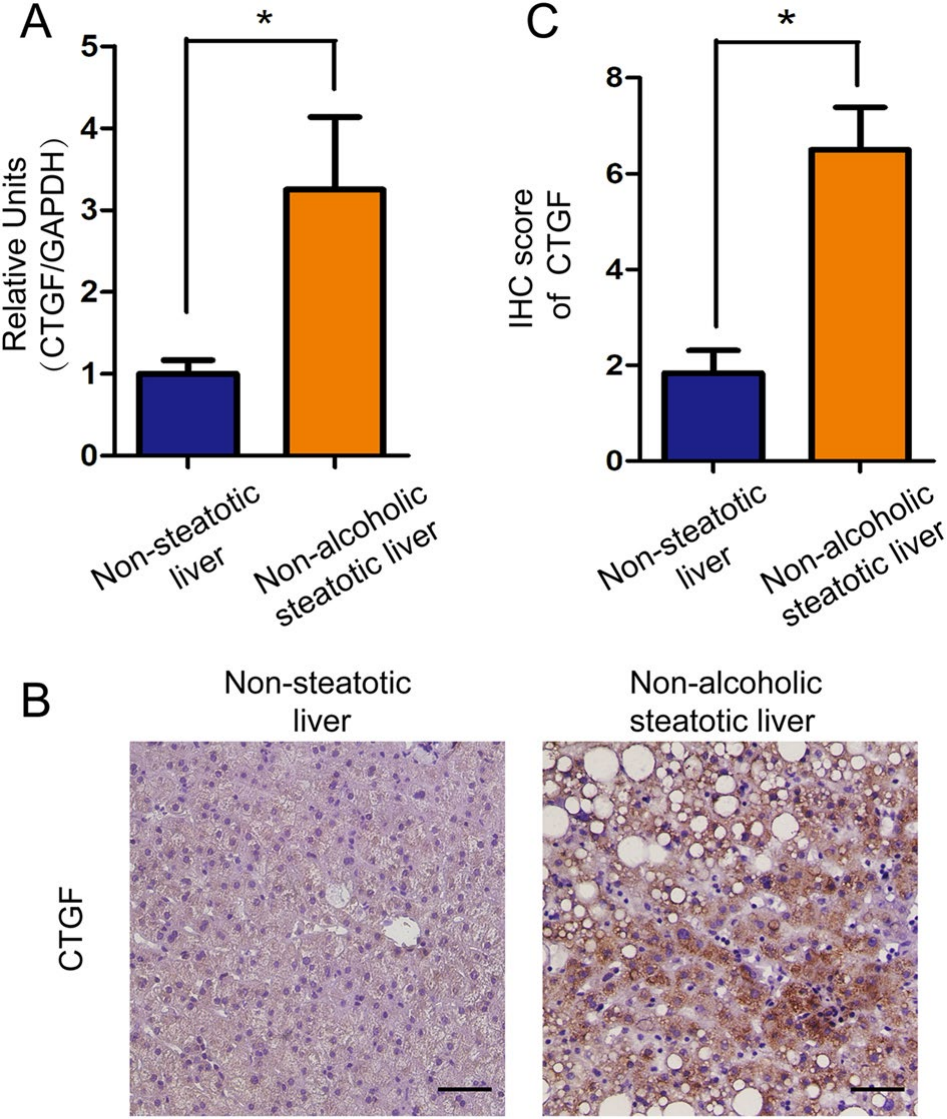
Expression of CTGF in the non-steatotic (n = 6) and non-alcoholic steatotic liver (n = 6) after brain death in humans. (**A**) CTGF expression was down-regulated in the non-steatotic liver and up-regulated in the non-alcoholic steatotic liver after brain death as determined by RT-qPCR (*P < 0.05). (**B**) Expression of CTGF in the non-steatotic liver and in the non-alcoholic steatotic liver after brain death by IHC (Scale bar = 20 μm, ×200 magnification) and (**C**) its semi-quantitation (*P < 0.05).

### Human samples

The human study was conducted under ethical approval of the Research Committee of the Affiliated Hospital of Zhengzhou University (No. 2018-KY-73), and all patients or their family members provided the informed consent. The human study was performed in accordance with the ethical principles of Declaration of Helsinki^21^. All donors were from the intensive care unit of the hospital and were selected following the national organ donation protocol^22^ (Supplementary Table 1 and Supplementary Fig. 1). No organs or tissues were procured from prisoners. Human liver and blood samples for research purposes were collected from brain-dead donors at the First Affiliated Hospital of Zhengzhou University.

All the patients in this study were grouped into two cohorts. In cohort 1, a number of 6 brain-dead donors with non-steatosis and 6 brain-dead donors with non-alcoholic steatosis were enrolled to compare the intrahepatic CTGF expression. In cohort 2, 27 brain-dead donors with NAFLD and non-steatosis were used to evaluate the correlation between CTGF expression and NAFLD activity in livers. The clinic-pathological characteristics were summarized in Supplementary Table 1 and the diagnosis was confirmed from the pathology reports. Alcoholic fatty liver disease and virus infection were ruled out in the cohorts.

### Cell culture and an in vitro hypoxia-reoxygenation model

Human HSC cell line, LX2, was obtained from American Type Culture Collection (Manassas, USA) and cultured in DMEM with 1% penicillin/streptomycin and 10% fetal bovine serum at 37 °C. LX2 cells were incubated under hypoxia (1% oxygen, 5% carbon dioxide and 94% nitrogen) in the hypoxia chamber (Galaxy 48 R, Eppendorf, Hamburg, Germany) for 3 h, followed by reoxygenation (hypoxia*/* reoxygenation) under the normoxic conditions (21% oxygen, 5% carbon dioxide and 74% nitrogen) for 6 h to mimic activation of HSCs under ischemia–reperfusion injury^23^.

To evaluate the dynamic change after hypoxia*/*reoxygenation, LX2 cells were harvested at 0 h, 1 h, 2 h, 4 h and 6 h after normoxic conditions for RT-qPCR and Western blot analyses (Supplementary Fig. 1).

To investigate the roles of YAP in regulation of CTGF expression, YAP siRNA or YAP function inhibitor (Verteporfin, 2 μM, s1786, Selleck Chemicals, Houston, USA) was incubated before hypoxia to observe the roles of YAP in the regulation of CTGF expression LX2 cells.

### siRNA transfection

YAP siRNA (5′ to 3′ CCACCAAGCUAGAUAAAGAdTdT) and control (catalog number AM17110) were produced by GenePharma (Shanghai, China). The transfection into the LX2 cells was carried out with Lipofectamine RNAiMAX Reagent (Cat.13778-075, Invitrogen, Waltham, USA) in Opti-MEM Medium (Gibco, Life Technologies**,** Rockville, USA) according to the manufacturer’s protocol.

### Gene expression analysis and reverse transcription-quantitative polymerase chain reaction (RT-qPCR)

Total RNA was isolated from liver tissue samples or cells using RNAiso reagent (Takara, Shiga, Japan). Specific primers for RT-qPCR used in the study (Supplementary Table 2) were designed and synthesized by Invitrogen (Waltham, USA). Reverse transcription and RT-qPCR (RR047A and RR840A, Takara*,* Shiga, Japan*)* were performed according to the manufacturer's protocol.

### Western blot

Primary antibodies used were anti-CTGF (1:1000, sc-101586, Santa Cruz, Dallas, USA), p-YAP (1:1000, #13008, Cell Signaling Technology, Beverly, USA), YAP (1:1000, #14074, Cell Signaling Technology, Beverly, USA), Smad2/Smad3 (1:1000, #5678, Cell Signaling Technology, Beverly, USA), p-Smad2/Smad3 (1:1000, #8828, Cell Signaling Technology, Beverly, USA), GAPDH (1:5000, 60004-1-Ig, Proteintech, Rosemont, USA). Immunoreactivities were visualized by secondary horseradish peroxidase-conjugated rabbit (1:2000, SA00001-2, Proteintech, Rosemont, USA), mouse (1:2000, SA00001-1, Proteintech, Rosemont, USA**)**.

### Hematoxylin–eosin (HE) staining and evaluation of NAFLD activity

Liver tissue were fixed in 4% paraformaldehyde in phosphate-buffered, embedded in paraffin wax, and stored at 4 °C. HE staining was performed as before^24^, and degree of steatosis (macrovesicular steatosis) and NAFLD activity were evaluated by the pathologist. Severity of steatosis was graded as non-steatotic liver donors (steatosis < 5%), mild steatosis (steatosis 5%-20%), moderate steatosis (21%-40%) and severe steatosis (> 40% and fibrosis). The scoring system of NAFLD activity was comprised of 4 histological features: steatosis (0–3), lobular inflammation (0–2), hepatocellular ballooning (0–2), and fibrosis (0–4) evaluated semi-quantitatively^25^. NAFLD activity score of ≥ 5 corresponded to a high-activity NAFLD, a score of 3–4 corresponded to a moderate-activity NAFLD, and a score of less than 3 corresponded to a non/low-activity NAFLD.

### Immunohistochemistry (IHC) and immunofluorescence

Primary and secondary antibodies against CTGF (1:50, sc-101586, Santa Cruz, Dallas, USA), α-SMA (1:100, Ab5694, Abcam, Cambridge, UK), p-YAP(1:100, Ser127 , #13008, Cell Signaling Technology, Beverly, USA), YAP (1:100, #14074, Cell Signaling Technology, Beverly, USA), transforming growth factor beta 1 (TGF-β1, 1:50, 21898-1-AP, Proteintech, Rosemont, USA), secondary against rabbit IHC kit (1:100; cat. no. SPN-9001; Beijing Zhongshan Golden Bridge Biotechnology Co., Ltd.), against mouse IHC kit (1:100; cat. no. SPN-9002; Beijing Zhongshan Golden Bridge Biotechnology Co., Ltd.), anti-Rabbit Cy3-conjugated Affinipure Goat (1:100, SA00009-1, Proteintech, Rosemont, USA), anti-Mouse 488-conjugated Affinipure Goat (1:100, SA00009-2, Proteintech, Rosemont, USA) were used in the immunostaining assays. Olympus IX71 microscope (Olympus Corporation,Tokyo, Japan) was used to observe the slides.

### Enzyme-linked immunosorbent assay (ELISA)

Human sera in cohort 2 were obtained, and the CTGF levels were determined with human CTGF SimpleStep ELISA Kit (ab245716, Abcam, Cambridge, UK) according to the manufacturer’s instructions.

### Statistical analysis

Values are given as means with standard deviation (SD). Differences in groups were analyzed by the two*-*tailed unpaired Student*’s* test or one-way ANOVA. The association analysis of CTGF expression with steatosis degree was employed with non-parametric (Spearman) correlation analysis. The statistical tests were employed by using SPSS version 21.0 (IBM, Armonk, New York, USA), and the results for *t* test, ANOVA and Spearman correlation were denoted as *t*,* F* and correlation coefficient. A probability level of less than 5% (*P* < 0.05) was considered statistically significant.

## Results

### CTGF expression was down-regulated in the non-steatotic liver after brain death in humans

Our previous study indicated that CTGF expression was downregulated in non-steatotic liver from brain-dead donors by RNA-sequencing (Supplemental Fig. 2 and 3). To compare the expression of CTGF in non-steatotic and non-alcoholic steatotic liver after brain death, we measured the CTGF in both mRNA and protein levels in non-steatotic and non-alcoholic steatotic liver from BDD donors. In human cohort 1, intrahepatic CTGF mRNA and protein expression were significantly down-regulated in the non-steatotic liver, whereas it was highly up-regulated in the non-alcoholic steatotic liver after brain death as determined by RT-qPCR (*t* = 2.483, *P* = 0.032, Fig. 1A) and IHC (*t* = 4.641, *P* = 0.001) (Fig. 1B,C).

**Figure 2:**
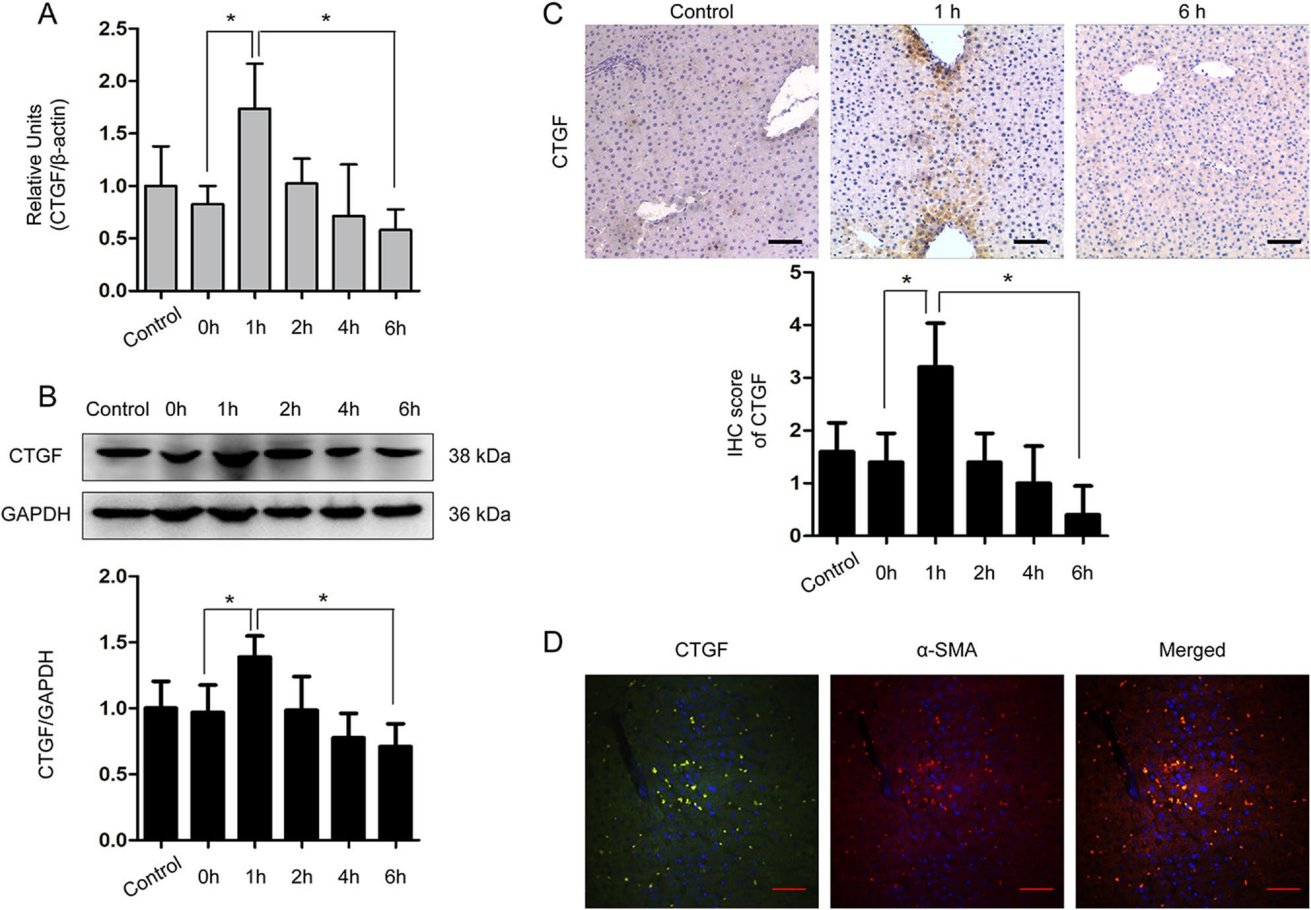
Expression dynamics of CTGF in the non-steatotic liver from the rat brain death model (n = 5 for each time point)*.* Dynamic expression of CTGF in the non-steatotic liver from the rat brain death model determined by (**A**) RT-qPCR, (**B**) WB, and (**C**) IHC (*P < 0.05 compared with 0 h). (**D**) Double immunofluorescence of CTGF (green) and α-SMA (red) in the rat liver 1 h after brain death (scale bar = 20 μm, ×200 magnification). The shame-operated rats were regarded as control.

**Figure 3:**
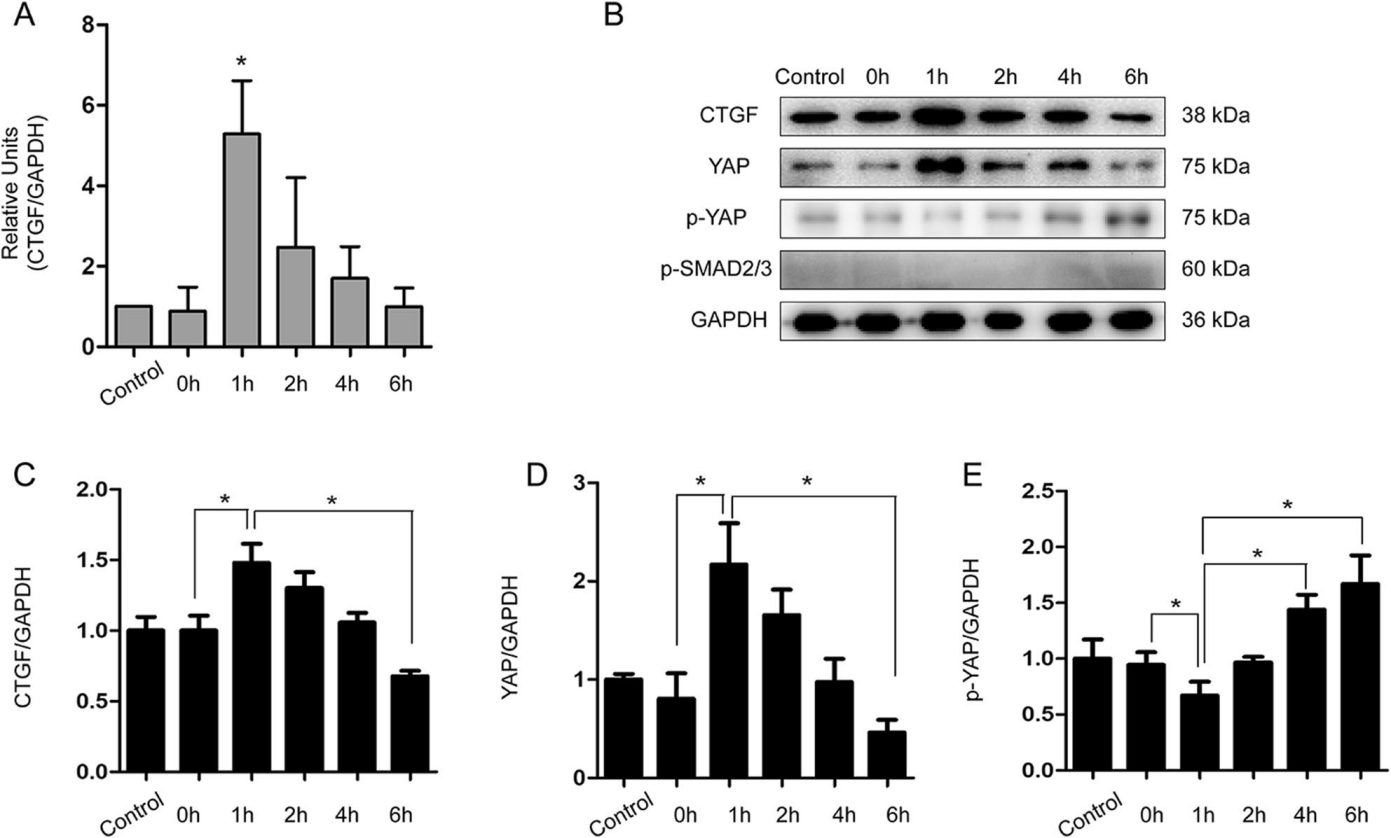
LX2 cells treated with hypoxia for 3 h and reoxygenation for different time with untreated LX2 cells as control. (**A**) Dynamics in the expression of CTGF mRNA in LX2 cells under hypoxia/reoxygenation (*P < 0.05). (**B**) Dynamic expressions of CTGF, YAP, p-YAP and p-SMAD2/3 in LX2 cells under hypoxia and reoxygenation culture conditions by WB and their quantifications (**C**–**E)** (*P < 0.05).

### Time-course analysis revealed intrahepatic CTGF expression in the non-steatotic liver after brain death

We further explored the dynamic expression of CTGF in the non-steatotic liver from a well-described rat model of brain death. The CTGF expression significantly increased in the rats (n = 5 for each time point), reaching the maximum at 1 h after induction of brain death, and then decreased gradually after 2 h until becoming significantly down-regulated at 6 h after brain death as determined by RT-qPCR (*F* = 8.105, *P* = 0.001, Fig. 2A), WB (*F* = 7.033, *P* = 0.001, Fig. 2B), and IHC (*F* = 10.950, *P* = 0.001, Fig. 2C).

To investigate the cellular origin of CTGF expression under brain death, hepatic cellular markers were detected alongside CTGF in tissue samples collected at different time points after brain death induction by double immunofluorescence. The results demonstrated that CTGF was co-expressed with α-SMA-positive cells at 1 h after brain death (Fig. 2D), indicating that intrahepatic CTGF was mainly regulated by active HSCs in the non-steatotic liver after brain death.

HSC undergoes activation and regulates tissue repair and after liver injury^26^. Next we sought to determine if CTGF expression associates with HSC activation. To this end, we cultured human HSC cell line LX2 and treated the cells with a hypoxia*/*reoxygenation procedure to mimic the activation of HSCs in the microenvironment of brain death. We found that CTGF expression was transiently up-regulated at 1 h after reoxygenation, and then decreased at 2 h after reoxygenation as determined by RT-qPCR (*F* = 8.686, *P* = 0.001; Fig. 3A) and WB (*F* = 6.913, *P* = 0.001; Fig. 3B,C), indicating a similar response and regulation of CTGF in HSCs as found in the brain-dead rat model.

### Intrahepatic CTGF expression in the non-steatotic liver was regulated through YAP under brain death

CTGF expression in activated HSCs are under regulation of either TGF-β pathway or Hippo/YAP^27,28^. To clarify the mechanism of CTGF down-regulation in the non-steatotic liver after brain death, we used transcriptome analysis from the non-steatotic liver samples of human brain-dead donors^18^. Kyoto Encyclopedia of Genes and Genomes (KEGG)^29^ enrichment analysis indicated that CTGF was correlated with the Hippo/YAP pathway and not with the TGF-β pathway (Supplementary Fig. 2 and 3) in the non-steatotic liver under brain death.

As described above, we found that CTGF expression was significantly down-regulated in the non-steatotic liver under brain death. Thus, we proposed that the down-regulation of CTGF could be resulted from inactivation of Hippo/YAP pathway in HSCs cells of the non-steatotic liver of BBDs. To assess this posibility, we measured YAP expression in cultured LX2 cells under hypoxia*/*reoxygenation treatment. We found that YAP expression was up-regulated cultured under hypoxia*/*reoxygenation at 1 h after reoxygenation, and was then subsequently down-regulated after 2 h, in agreement with the pattern of CTGF expression (*F* = 23.900, *P* = 0.001; Fig. 3B,D). In contrast, the p-YAP expression level decreased at 1 h, and increased at 4 h and 6 h after reoxygenation as determined by WB (*F* = 16.820, *P* = 0.001; Fig. 3B,E). However, the level of p-SMAD2/3 did not increase during this process (Fig. 3B).

**Figure 4:**
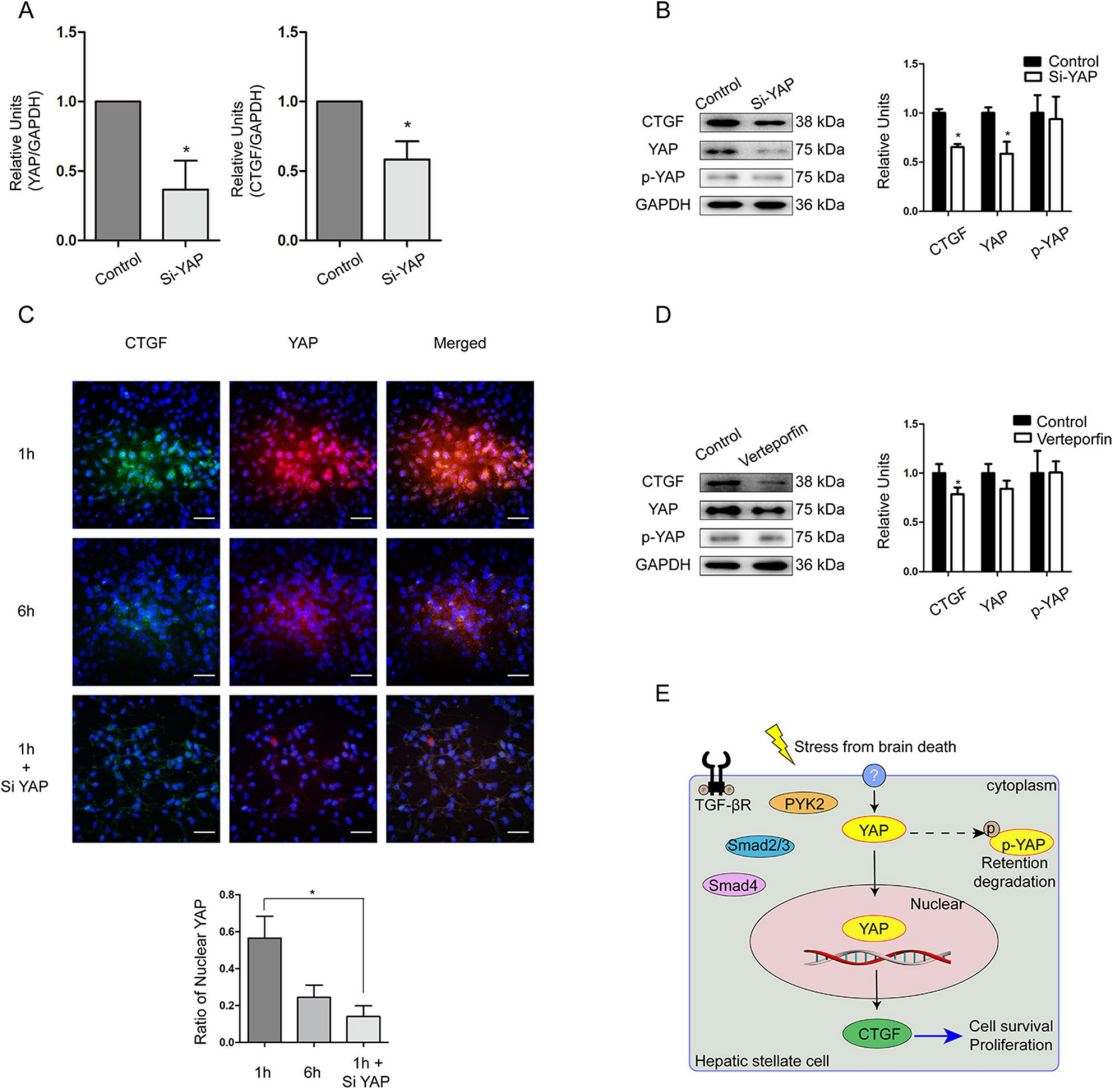
Regulation of CTGF through YAP in LX2 cells treated with Si-RNA or YAP inhibitor Verteporfin under hypoxia for 3 h and reoxygenation for 1 h. LX2 cells treated with vehicle of Si-RNA or Verteporfin were regarded as control. (**A**) Changes of YAP and CTGF mRNAs in LX2 cells after YAP-siRNA treatment under hypoxia/reoxygenation determined by RT-qPCR (*P < 0.05 between siRNA and control). (**B**) Changes of CTGF, YAP, and p-YAP protein levels after YAP-siRNA treatment in LX2 cells determined by WB (*P < 0.05 between siRNA and control). (**C**) Immunofluorescence staining of CTGF (green) and YAP (red) in LX2 cells under hypoxia/reoxygenation; DAPI (blue) was used for nuclear staining (scale bar = 20 μm, ×200 magnification) and semi-quantitation of nuclear YAP (*P < 0.05). (**D**) Changes of CTGF, YAP, and p-YAP expression in LX2 cells after treatment of the YAP inhibitor Verteporfin under hypoxia/reoxygenation (*P < 0.05). (**E**) Schematic of the proposed mechanism of CTGF regulation in the non-steatotic liver after brain death.

To further verify the underlying regulation mechanism, we used siRNA technology to silence YAP mRNA in LX2 cells. After knocking down YAP expression, CTGF expression was significantly down-regulated at 1 h after reoxygenation as determined by RT-qPCR (*t* = 5.517, *P* = 0.031; Fig. 4A) and WB (*t* = 11.776, *P* = 0.001; Fig. 4B). Immunofluorescence analysis further showed that the percentage of nuclear YAP was higher at 1 h, and then decreased at 6 h after reoxygenation (*t* = 5.317, *P* = 0.002; Fig. 4C), with a further decrease of nuclear YAP observed in siRNA-treated LX2 cells at 1 h after reoxygenation (*t* = 7.243, *P* = 0.001; Fig. 4C). This effect was confirmed after treatment with the YAP-specific inhibitor verteporfin under hypoxia*/*reoxygenation, in which CTGF expression down-regulated at 1 h after reoxygenation as determined by WB (*t* = 3.195, *P* = 0.019; Fig. 4D). These data suggested that CTGF expression HSCs of non-steotic liver is dependent on Hippo/YAP pathway. The proposed mechanism of regulation of CTGF in the non-steatotic liver after brain death is schematically illustrated in Fig. 4E^30^.

### Intrahepatic CTGF expression in the non-alcoholic steatotic liver after brain death might be dependent on the TGF-β pathway

**Figure 5:**
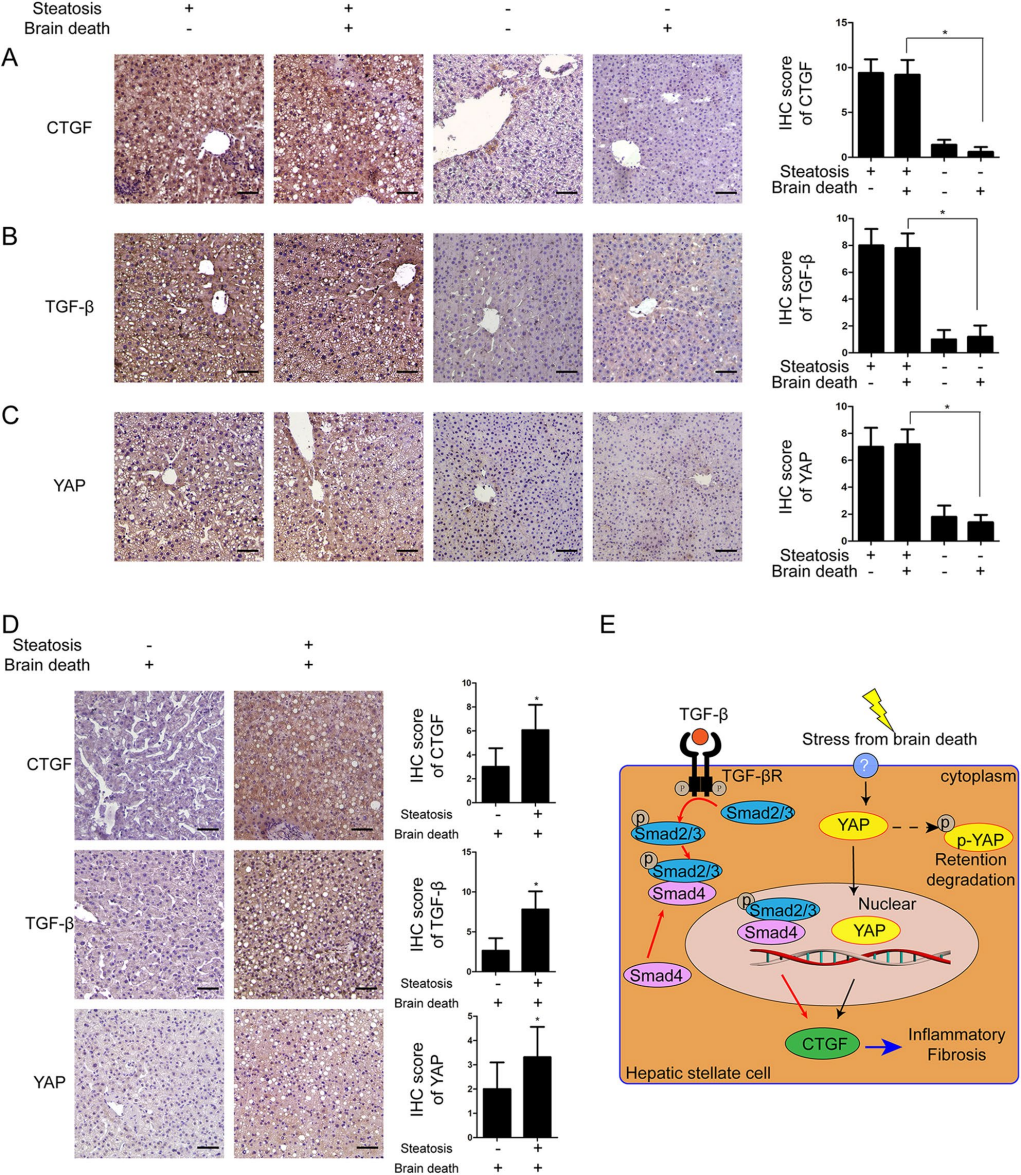
Expression of CTGF, YAP and TGF-β in non-steatotic liver and non-alcoholic steatotic liver under brain death. Expression of (**A**) CTGF, (**B**) TGF-β, and (**C**) YAP in the non-steatotic liver and non-alcoholic steatotic liver before and after brain dead in rats; n = 5 per group, scale bar = 20 μm, ×200 magnification (*P < 0.05 between the non-steatotic liver and non-alcoholic steatotic liver after brain death). (**D**) Expression of CTGF, TGF-βand YAP in human non-steatotic and non-alcoholic steatotic liver tissues (scale bar = 20 μm, ×200 magnification, n = 6). (**E**) Schematic of the proposed mechanism of CTGF regulation in the non-alcoholic steatotic liver after brain death.

IHC analysis showed that CTGF was highly overexpressed in the steatotic liver both before and after brain death in rats without a significant difference (n = 5 for each group, *t* = 11.103, *P* = 0.001; Fig. 5A). Furthermore, TGF-β and YAP were also overexpressed in the non-alcoholic steatotic liver after brain death in rats (n = 5 for each group, *t* = 10.707 and 4.333, respectively, *P* = 0.001 and 0.003; Fig. 5B,C).

In humans, CTGF, TGF-β and YAP were overexpressed in the non-alcoholic steatotic liver after brain death as determined by IHC (Fig. 5D). These results supported that overexpression of CTGF in the non-alcoholic steatotic liver, aggraved by brain death^14,15,17^, might be mainly mediated through the TGF-β pathway (Fig. 5E)^31^.

Instead, the expressions of TGF-β in the non-steatotic liver in rats were not significantly changed after brain death by RT-qPCR (n = 5 for each group, *F* = 0.380, *P* = 0.858) (Supplementary Fig. 4) and IHC (n = 5 for each group, *t* = 0.408, *P* = 0.694) (Fig. 5, Supplementary Fig. 4).

### CTGF expression was correlated with NALFD activity in the livers of brain-dead donors

**Figure 6:**
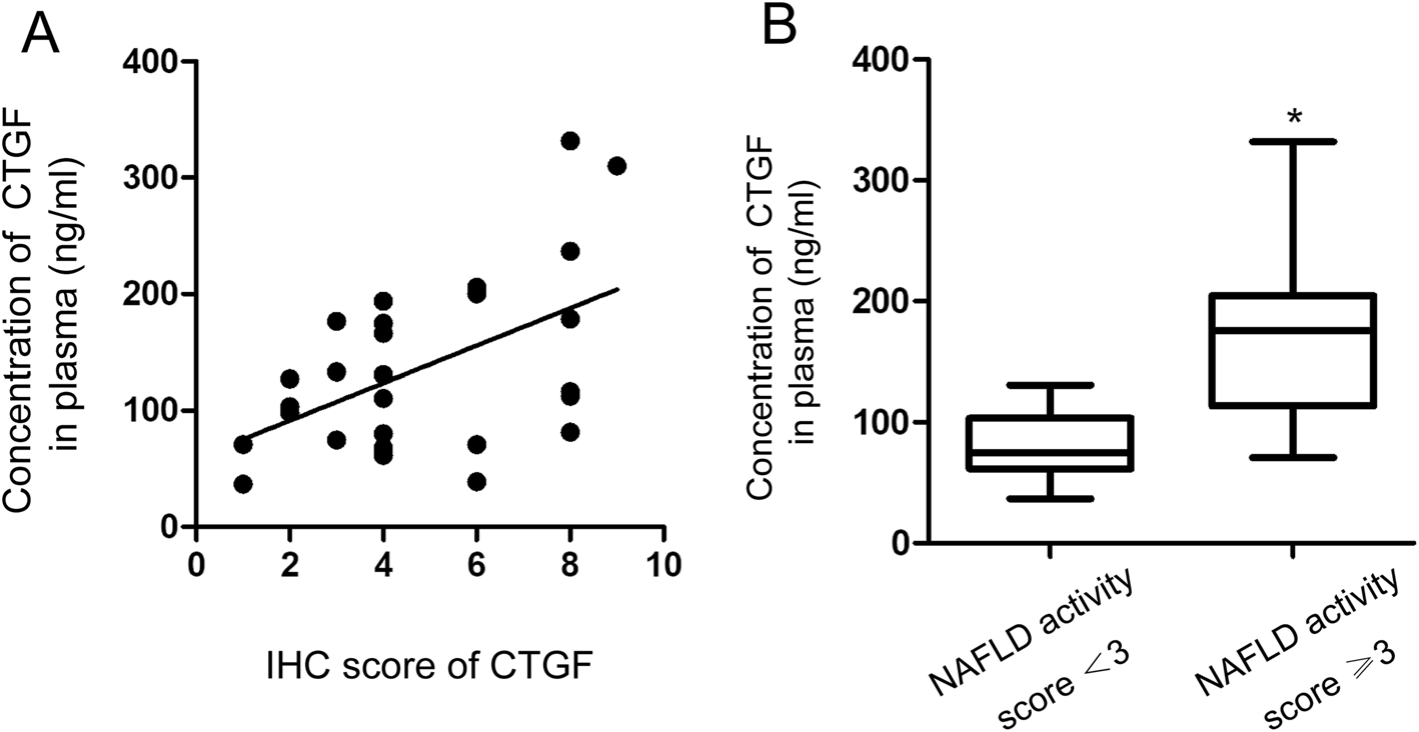
Correlation between CTGF expression and NAFLD activity in humans. (**A**) Correlation between systemic CTGF levels and semi-quantitative immunoreactivity scores of intrahepatic CTGF in cohort 2 (n = 27). (**B**) Systemic CTGF levels was higher in moderate and high NAFLD activity group (NAFLD activity score ≥ 3, n = 16) compared with no or no or low NAFLD activity group (NAFLD activity score ˂3, n = 11) (*P < 0.05).

Given the different expression and regulation patterns of CTGF between the non-steatotic and non-alcoholic steatotic liver in rats under brain death, we further explored whether CTGF expression might predict the degree of NAFLD activity under brain death using samples from human brain-dead donors in cohort 2. The serum CTGF concentrations determined by ELISA and intrahepatic CTGF expression levels determined by IHC with semi-quantitative immunoreactivity scores were positively correlated (Spearman correlation coefficients = 0.442, *P* = 0.021; Fig. 6A). In addition, the serum CTGF concentration was positively correlated with the NAFLD activity score (Spearman correlation coefficients = 0.499, *P* = 0.008)*.*

The concentrations of CTGF in cases of moderate (NAFLD score of 3–4) and high (NAFLD score ≥ 5) NAFLD activity (n = 16) were higher than those in cases with no or low NAFLD activity (NAFLD score < 3) (n = 11) (*t* = 3.971, *P* = 0.001; Fig. 6B)*.* The area under the receiving operator characteristic curve for the prediction of moderate/high-activity NAFLD (NAFLD score ≥ 3) was 0.903 (95% confidence interval 0.791–1.000). A cut-off value of 106.93 μg/L, with a sensitivity of 87.5% and a specificity of 81.8%, had a positive predictive value of 87.5% and negative predictive value of 81.8%.

## Discussion

The severity of steatosis of a liver graft is typically evaluated according to histopathological changes from liver biopsy during graft procurement^10^. Liver grafts with mild steatosis can be safely used for liver transplantation; however, livers with severe (> 60%) steatosis and NASH are usually discarded owing to the increased risk of primary non-function after transplantation, and those with moderate (40–60%) steatosis have also been associated with decreased graft function and graft survival if transplanted^10^. Thus, identification of a non-invasive biomarker that can provide reliably good accuracy to predict the steatosis degree, NAFLD activity, and inflammation-related graft injury is urgently needed for improving liver graft assessment from a brain-dead donor^5,6^.

The findings from our previous study^18^ and the current study demonstrated that CTGF expression after brain death was down-regulated in the non-steatotic liver but was overexpressed in the non-alcoholic steatotic liver of rats and humans. The clinical diagnosis of brain death requires reconfirmation at 6–12 h after the first diagnosis. The acute-phase response during this period may provide biological information to elucidate the regulation mechanism of potential biomarkers, which have thus far been neglected. Using the rat model of brain death, we explored the dynamic expression of CTGF in the non-steatotic liver after the induction of brain death. CTGF levels significantly increased initially after brain death, reaching the maximum at 1 h, and then gradually decreased to become significantly down-regulated at 6 h after brain death induction. By contrast, there was no significant difference in CTGF expression in the steatotic liver before and after brain death in rats.

The further correlation analysis based on the human samples showed that NAFLD activity in the non-alcoholic steatotic liver was positively correlated with donor CTGF levels. Thus, measurement of the blood CTGF level by ELISA before liver graft procurement would provide a rapid method to reliably assess the steatosis grade and NAFLD activity from a potential donor with NAFLD. We found that a brain-dead donor with a CTGF blood level above 106.93 μg/L is a reliable cut-off to indicate the need for further testing during graft assessment. However, this cut-off value should be validated with additional clinical data.

Transcriptome and KEGG signaling analysis revealed that the Hippo/YAP signaling pathway might mediate the acute brain death-related injury in the non-steatotic liver through down-regulation of CTGF. This potential role of Hippo/YAP on CTGF regulation was further verified by treatment of YAP siRNA or a YAP inhibitor under in vitro hypoxia/reoxygenation in LX2 cells. Moreover*,* TGF-β overexpression was found after brain death in the non-alcoholic steatotic liver, confirming previous reports showing that the grade of NAFLD activity, including hepatic steatosis, inflammation and fibrosis, is correlated with TGF-β expression level^5,6^, and HSCs can mediate the progression of NAFLD through TGF-β^32–34^. Overall, these findings indicate that the YAP-CTGF axis in HSCs may be a key player modulating the acute liver injury caused by brain death in the non-steatotic liver, and TGF-β/CTGF may account for the major mechanism contributing to moderate/high NAFLD severity after brain death^35,36^. Thus, our findings suggested that in the brain death-related acute response, regulation of CTGF expression in HSCs is mediated by YAP in the non-steatotic liver, while in the CCl_4_-induced liver fibrosis and the non-alcoholic steatotic liver is still dominated by TGF-β or TGF-β induced proline-rich tyrosine kinase 2 pathway^35,37^. The effect of YAP in HSC activation and liver injury was evidenced by a recent study claiming that activation of YAP attenuates hepatic damage in liver ischemia–reperfusion injury^23^. The down regulation of YAP-CTGF pathway in non-steaotic liver under brain death provides an interfering target for perservation of liver graft of BDDs. Pharmacological activation of Hippo/YAP pathway may increase the expression of CTGF, which has protective function for liver injury.

One of the limitations of this study is the lack of genetic knockout animal models or in vivo virus-mediated gene transduction, which could provide more explicit causal evidence. However, brain death comprises a complex pathophysiological process involving a transient catecholamine storm after cerebral injury, leading to a subsequent catecholamine deficit^38^. Therefore, we employed the well-described brain death animal model with immune-component rodents without further gene interference, given that the effect of knockout or gene transduction on brain death in rats is still unclear. An additional limitation of the human study is the small number of patients, making it difficult to reliably detect CTGF expression and its correlation with NAFLD activity. Since few study reported identification of a non-invasive biomarker from the extended-criteria donor with severe (> 60%) steatosis and NASH, we managed to identify a non-invasive biomarker that may provide reliably good accuracy to predict the steatosis degree and NAFLD activity. The further studies with more patients are warranted to evaluate the clinical prognosis potential based on the current findings.

**Figure 7:**
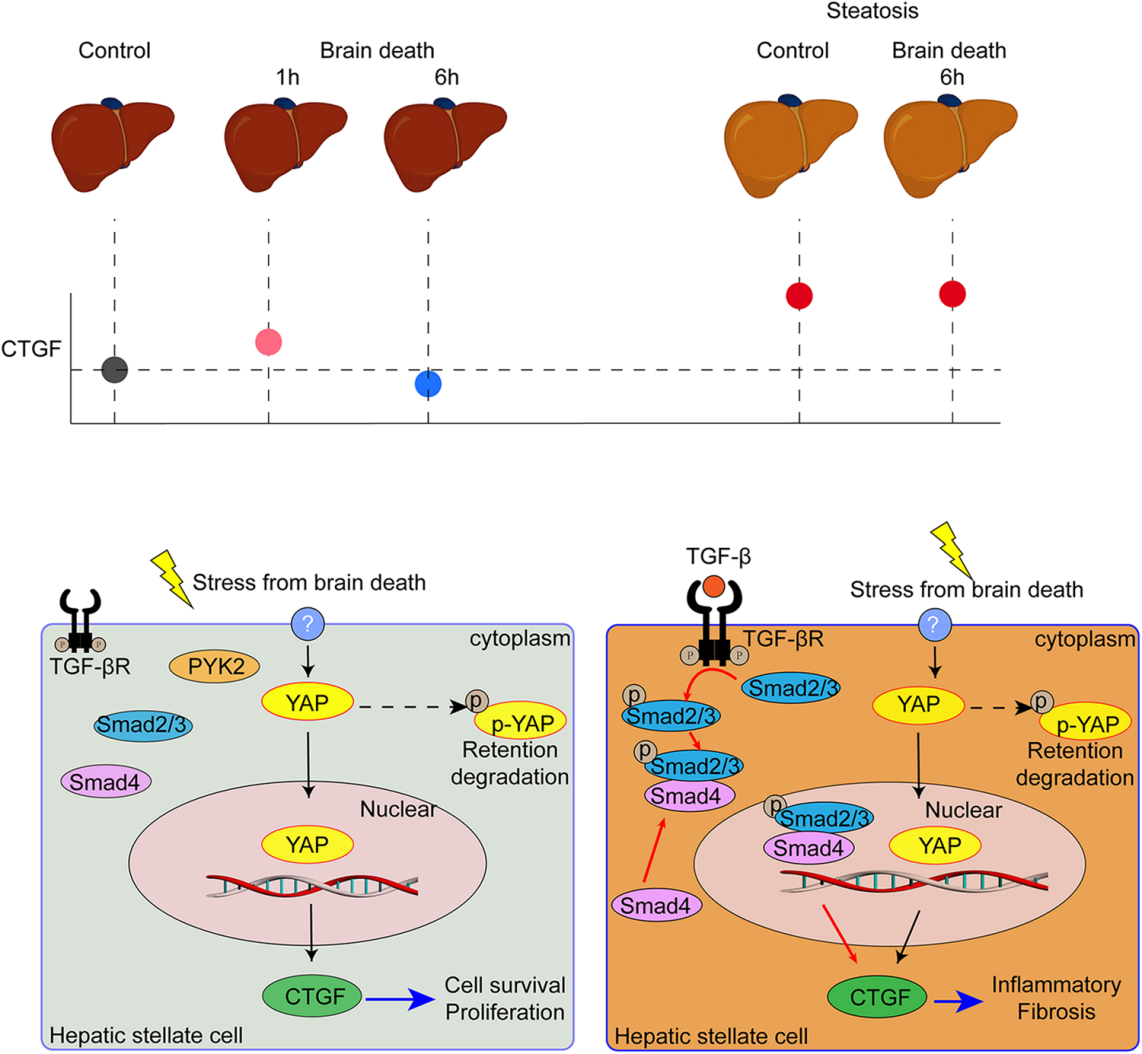
CTGF expression in hepatic stellate cells and its regulation in the non-steatotic liver and the non-alcoholic steatotic liver after brain death.

In conclusion, CTGF expression and regulation differ between nonsteatosis and nonalcoholic steatosis livers from BDDs. Down-regulation of CTGF could be considered as a potential biomarker to predict a non/mild steatotic liver from NAFLD donors, and CTGF up-regulation could indicate a risk of a moderate/severe steatotic liver that requires further examination prior to graft selection (Fig. 7).

## Supplementary Information

Supplementary Information.

## Abbreviations

BDD: Brain-dead donor
NAFLD: Nonalcoholic fatty liver disease
CTGF: Connective tissue growth factor
CCN: Cellular Communication Network Factor
TGF-β: Transforming Growth Factor Beta
YAP: Yes Associated Protein
NASH: Non-alcoholic steatohepatitis
ECM: Extracellular matrix
HSCs: Hepatic stellate cells
α-SMA: Alpha smooth muscle actin
