# Supplementary material for: Hepatic connective tissue growth factor expression and regulation differ between non-steatotic and non-alcoholic steatotic livers from brain-dead donor
Source: Sci Rep. 2021 Feb 16;11:3857. doi: 10.1038/s41598-021-83516-2 (PMC7886893; doi:10.1038/s41598-021-83516-2)

**Supplementary Material**

**Hepatic connective tissue growth factor expression and regulation differ between non-steatotic and non-alcoholic steatotic livers from brain-dead donor**

**Running Title:** Hepatic CTGF after DBD

Dong-Jing Yang, MD^1^**#**, Ji-Hua Shi, MD^1^ **#**, Zong-Ping Xia, PhD^2^, Wen-Zhi Guo, MD, PhD^1^, Mohammed Shakil Ahmed, PhD^3^, Shui-Jun Zhang, MD, PhD^1^*

1.Department of Hepatobiliary and Pancreatic Surgery, Henan Key Laboratory of Digestive Organ transplantation & Zhengzhou Key Laboratory for HPB diseases and Organ transplantation, The First Affiliated Hospital of Zhengzhou University, Zhengzhou University, Zhengzhou, China.

2. Translational Medicine Center, The First Affiliated Hospital of Zhengzhou University, Zhengzhou University, Zhengzhou, China.

3.Institute for Surgical Research, Department of Cardiology and Center for Heart Failure Research, Oslo University Hospital- Rikshospitalet and University of Oslo.

**#** The first two authors contributed equally to the work.

***Corresponding author**:

Shui-Jun Zhang, *Prof, MD*, *PhD*

Department of Hepatobiliary and Pancreatic Surgery, The First Affiliated Hospital of Zhengzhou University, Zhengzhou University.

Email: zhangshuijun@zzu.edu.cn

**Supplementary Material**

**Supplementary Figure 1**

Time points of collecting biopsy specimens.


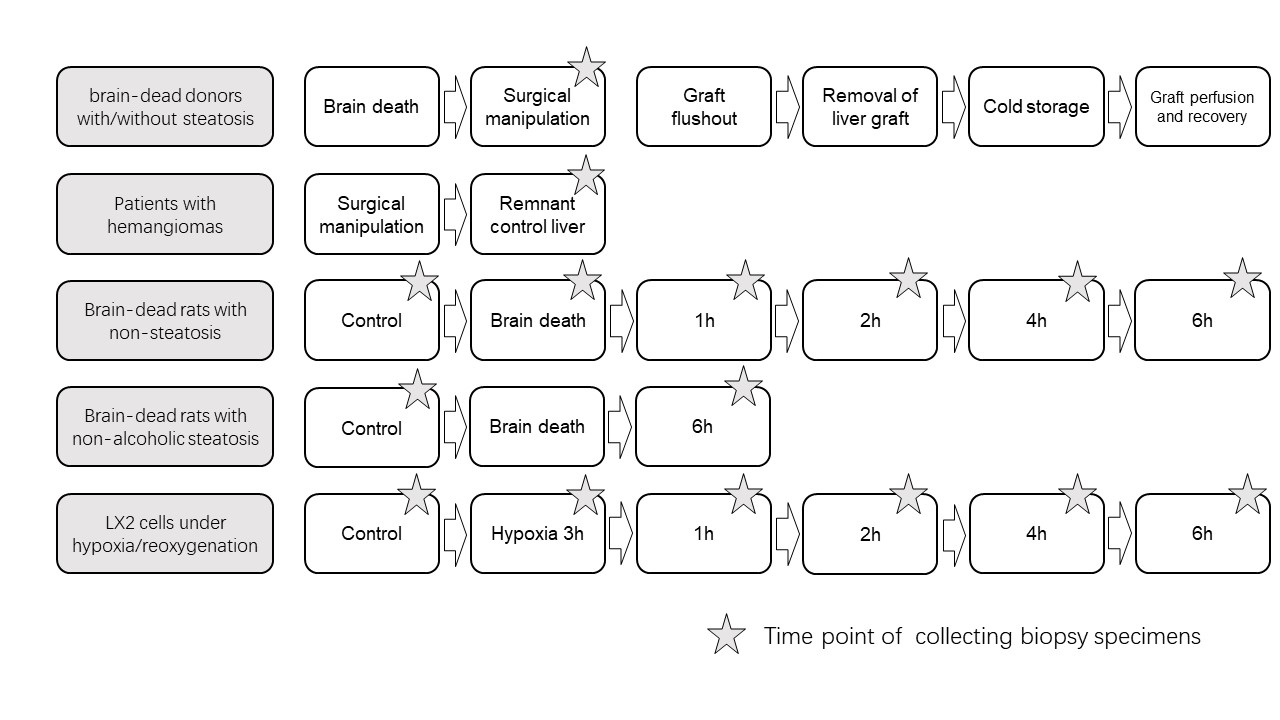


**Supplementary Material**

**Supplementary Figure 2**

Non-steatotic livers tissues from 3 brain-dead donors and 3 hepatic patients with hemangioma resection as control (without brain death) were used for RNA-sequencing.

**A.** Analysis of transcriptome showed that 4367 mRNAs were differentially expressed by *P* value<0.05 & |log2FC|>1. Among them, 1884 genes were upregulated and 2483 were downregulated.

**B.** The CTGF was found down-regulated in the non-steatotic liver from brain-dead donors compared with control with the fold change of 2.941 (*P*=0.009).

C. TGFβ in the non-steatotic liver from brain-dead donors were not significantly changed after brain death compared with control (*P*>0.05).


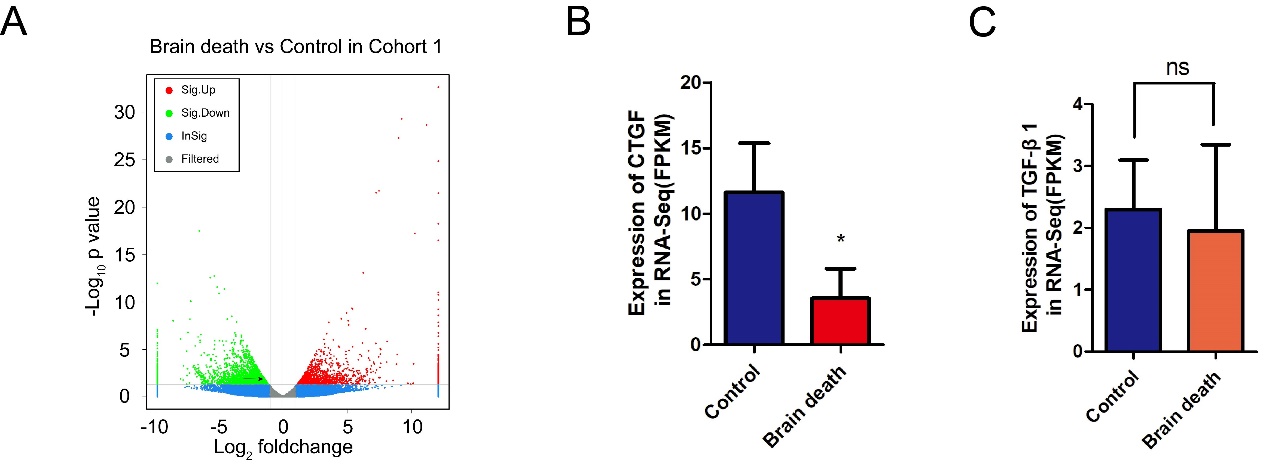


**Supplementary Material**

**Supplementary Figure 3**

Non-steatotic livers tissues from 6 brain-dead donors and 6 hepatic patients with hemangioma resection as control (without brain death) were used for validating the expression of CTGF.

**A** and **B.** The CTGF protein was found down-regulated in the non-steatotic liver from brain-dead donors compared with control determined by IHC (*P*=0.020).

**C** and **D**. TGFβ were not significantly changed after brain death in the non-steatotic liver from brain-dead donors compared with control determined by IHC (*P*>0.05).


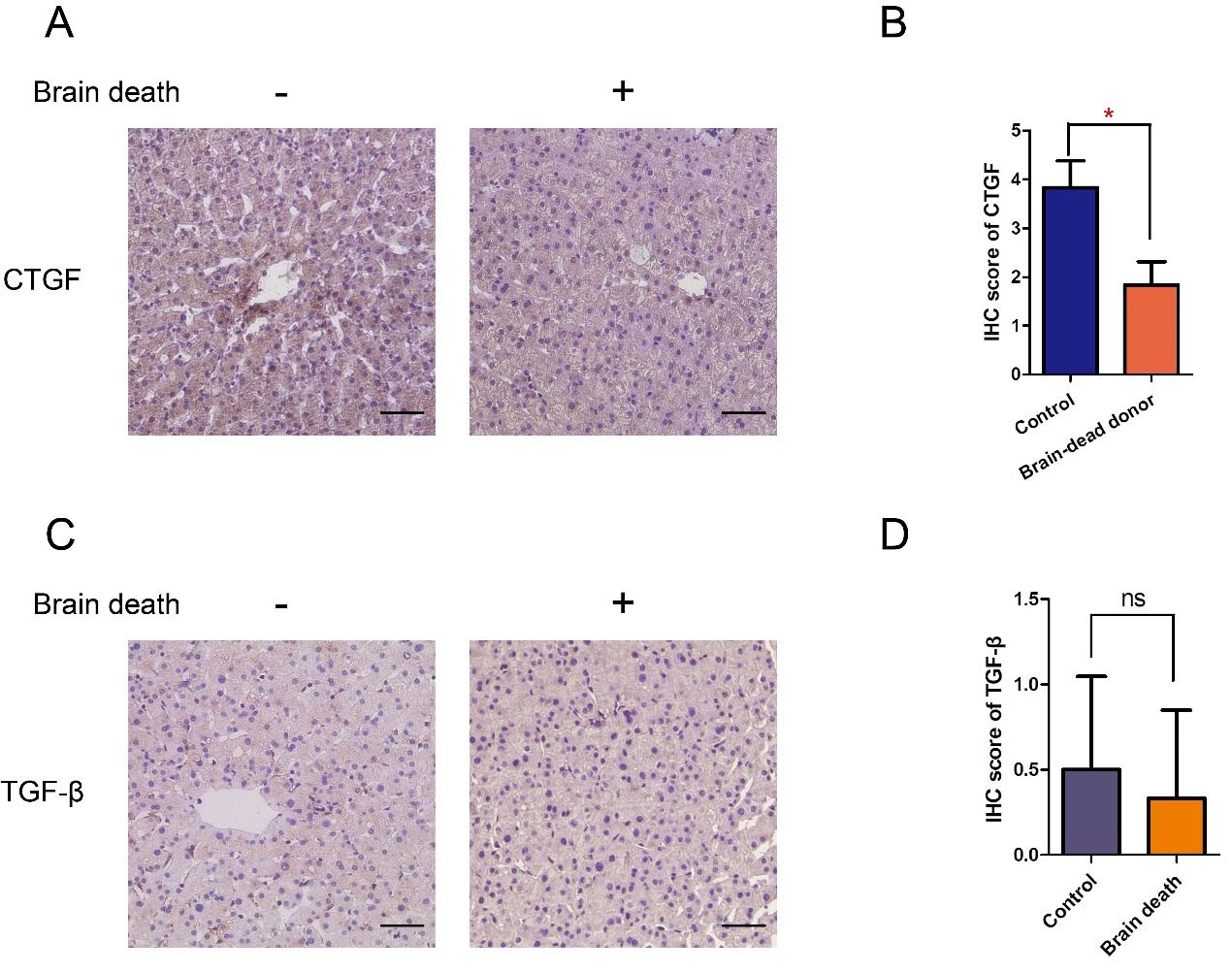


**Supplementary Material**

**Supplementary Figure 4**

Expression dynamics of TGF-β in the non-steatotic liver from the rat brain death model (n=5 for each time point). Dynamic expression of TGF-β was determined by (A) RT-qPCR, (B) IHC (scale bar = 20 μm, 200× magnification), and (C) its semi-quantitation. The shame-operated rats were regarded as control.


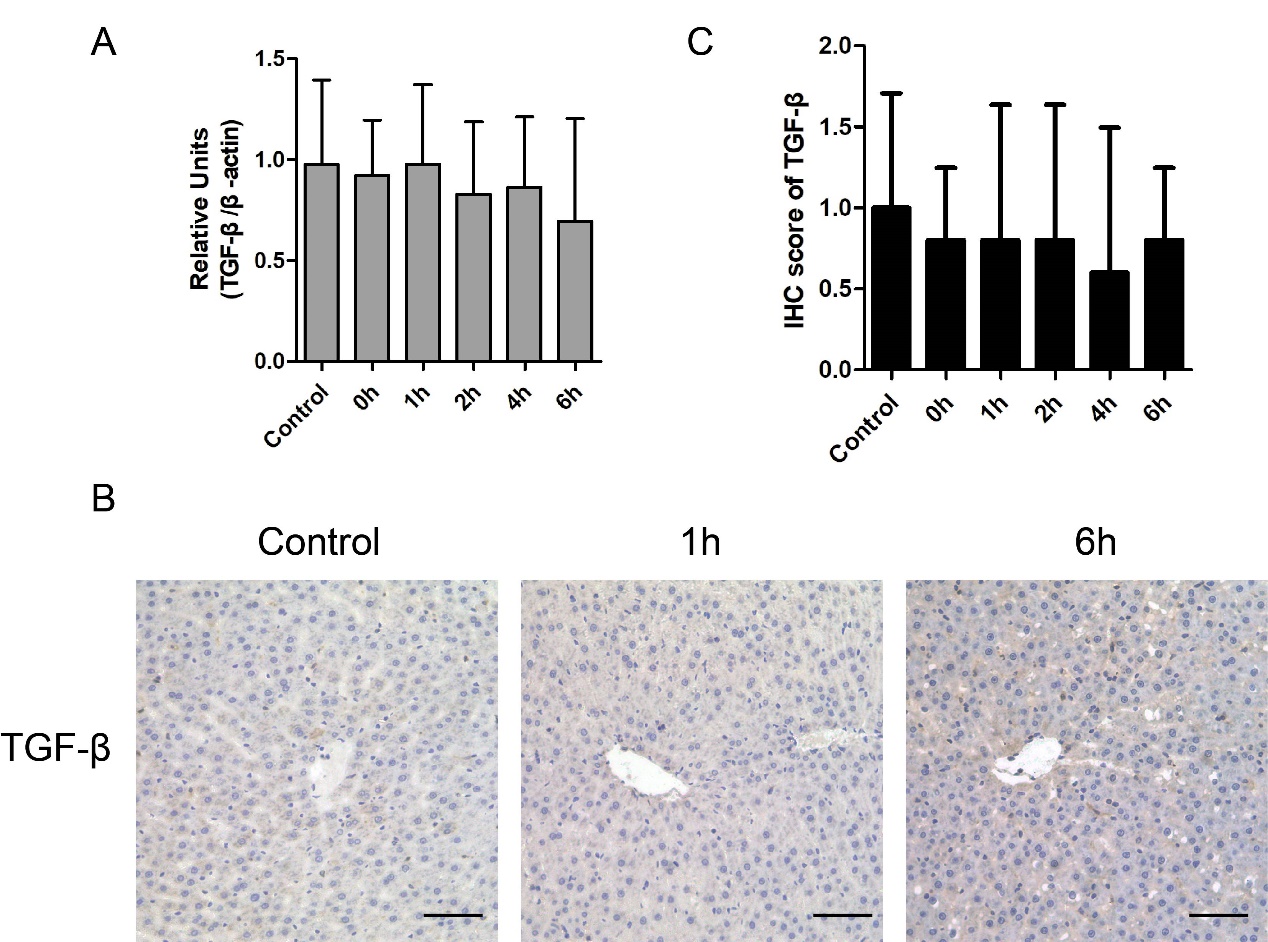


**Supplementary Material**

**Supplementary Table 1.**

**Donor factors for non-steatotic liver group and non-alcoholic steatotic liver group.**

**Cohort 1**

| Variable | non-steatotic  （n=6） | non-alcoholic steatotic （n=6） |
| --- | --- | --- |
| Donor gender |  |  |
| Male | 5 | 4 |
| Female | 1 | 2 |
| Donor age (years ± SD) | 38.5 ± 17.8 | 52.33 ±7.8 |
| ICU (hours ± SD) | 51.8 ± 19.7 | 49.7 ± 26.5 |
| Cardiac arrest (yes/no) |  |  |
| yes | 0 | 1 |
| no | 6 | 5 |
| Hypotensive period |  |  |
| Yes | 1 | 1 |
| no | 5 | 5 |
| Causes of Brain death |  |  |
| cebrovascular accident | 3 | 4 |
| traumatic brain injuries | 3 | 2 |
| Steatosis % | 0 ± 0 | 40 ± 14.1* |
| NAFLD score | 0 ± 0 | 4.3 ± 1.3* |

**Cohort 2**

| Variable | non-steatotic  (n=11, NAFLD score＜3) | non-alcoholic steatotic  (n=16, NAFLD score≥3) |
| --- | --- | --- |
| Donor gender |  |  |
| Male | 8 | 13 |
| Female | 3 | 3 |
| Donor age (years ± SD) | 41.9 ± 11.0 | 49.0 ±10.9 |
| ICU (hours ± SD) | 40.2± 15.8 | 46.8 ± 29 |
| Cardiac arrest | 1/11 | 1/16 |
| yes | 1 | 1 |
| no | 10 | 15 |
| Hypotensive period |  |  |
| Yes | 1 | 2 |
| no | 10 | 14 |
| Causes of Brain death |  |  |
| cebrovascular accident | 9 | 10 |
| traumatic brain injuries | 2 | 6 |
| Steatosis % | 7.3 ± 8.5 | 44.7 ± 14.6* |
| NAFLD score | 0.9 ± 0.8 | 4.8 ± 1.3* |

**P*＜0.05

**Supplementary Material**

**Supplementary Table 2**.

**Primer sequences for RT-PCR in the study**

| **RT-PCR primers** | **Sequences** | **Species** |
| --- | --- | --- |
| CTGF forward | CTTGCGAAGCT GACCTGGAAGA | human |
| CTGF reverse | CCGTCGGTACATACTCCACAGA |  |
| YAP forward | CCCTCGTTTTGCCATGAACC | human |
| YAP reverse | GTTGCTGCTGGTTGG AGTTG |  |
| GAPDH forward | GTCTCCTCTGACTTCAACAGCG | human |
| GAPDH reverse | ACCACCCTGTTGCTGTAGCCAA |  |
| CTGF forward | CACCGCACAGAACCACCACA | rat |
| CTGF reverse | GGCAGGCACAGGTCTTGATGAAC |  |
| β-actin forward | CCGAGATCTCACCGACTACCTCA | rat |
| β-actin reverse | TCAAAGTCCAGAGCGACATAGCA |  |

**P*＜0.05

**Supplementary Material**

**Gel scans from the main figures**

**Figure 2B**


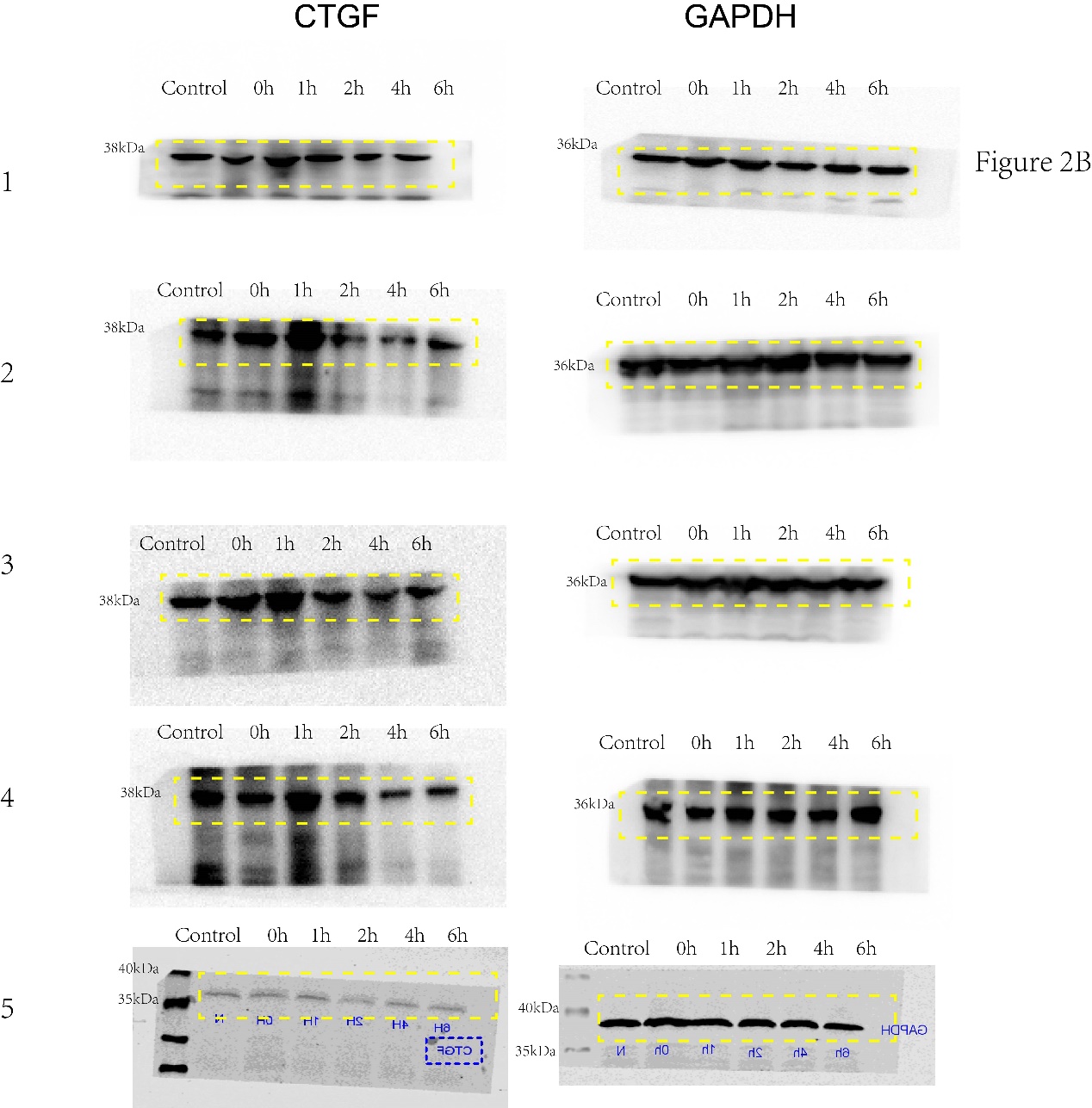


**Supplementary Material**

**Gel scans from the main figures**

**Figure 3B**


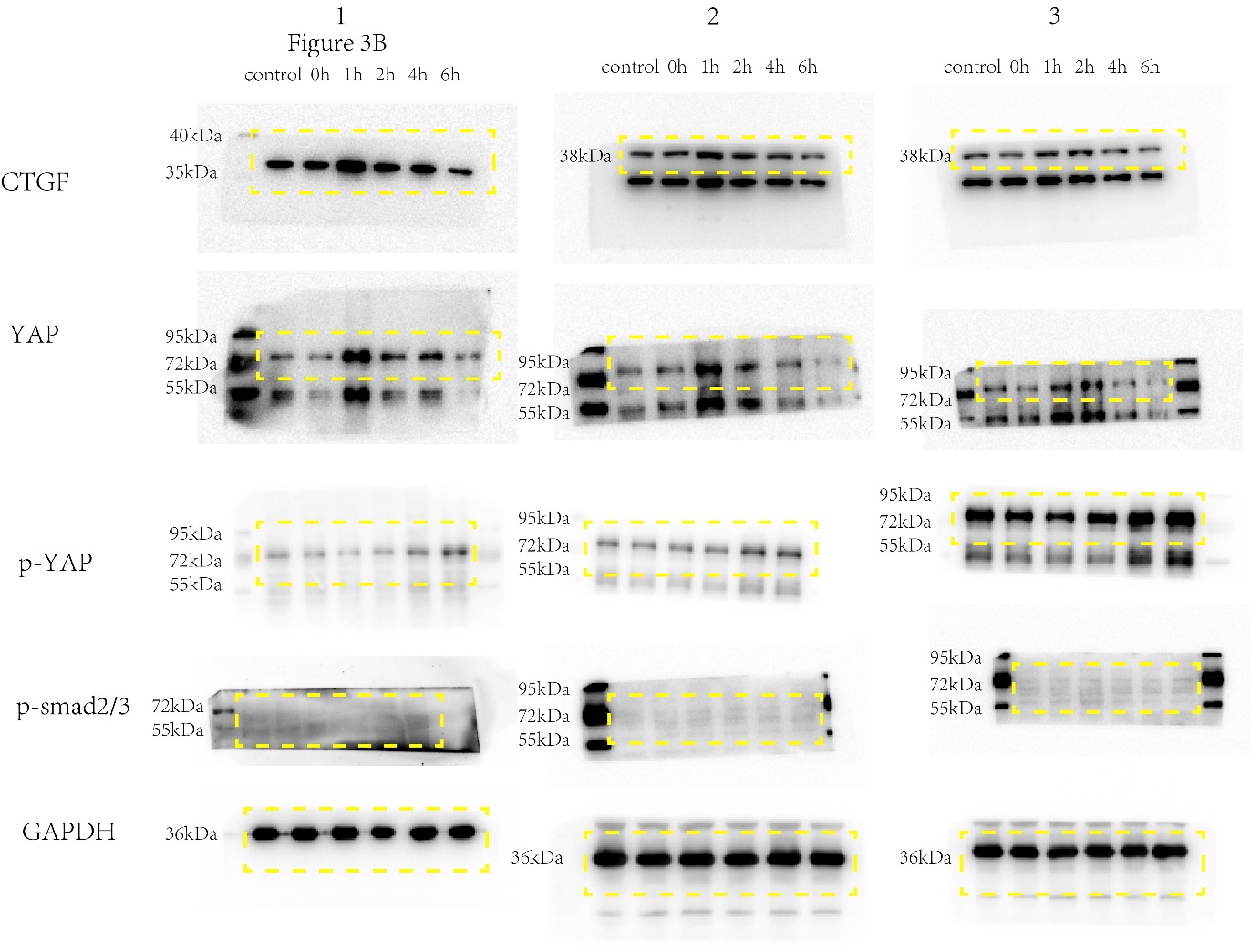


**Supplementary Material**

**Gel scans from the main figures**

**Figure 4B**


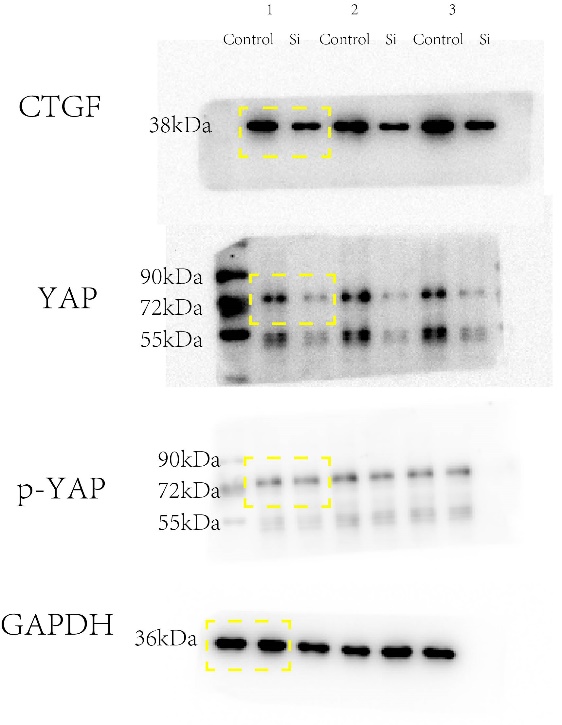


**Figure 4D**


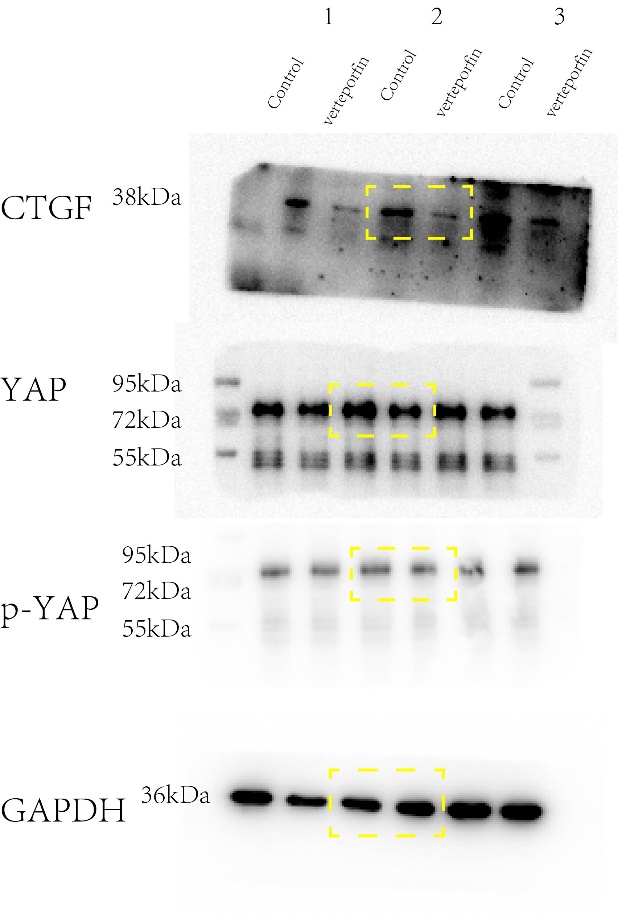

Supplement: Supplementary file 1 — Supplementary Information. [file 41598_2021_83516_MOESM1_ESM.docx]
